# Evaluating the Role of STAT3 in CD4^+^ T Cells in Susceptibility to Invasive Aspergillosis

**DOI:** 10.1128/IAI.00035-21

**Published:** 2021-04-16

**Authors:** Wajiha Gohir, Lisa McTaggart, Julianne V. Kus, Tony Mazzulli, Deepali Kumar, Atul Humar, Shahid Husain

**Affiliations:** a Transplant Infectious Diseases, Ajmera Transplant Center, University Health Network, University of Toronto, Toronto, Canada; b Public Health Ontario, Toronto, Canada; c Department of Laboratory Medicine and Pathobiology, University of Toronto, Toronto, Canada; d Department of Microbiology, Mount Sinai Hospital/University Health Network, Toronto, Canada; Tulane School of Medicine

**Keywords:** STAT3, invasive aspergillosis, T cells, immunosuppression

## Abstract

We aimed to determine whether T cell-specific STAT3 deletion influences the immune response to *Aspergillus* in the immunosuppressed context in CD4*^Stat3^*^−/−^ mice. Immunosuppressed and nonimmunosuppressed CD4*^Stat3^*^−/−^ mice and littermate Stat3^flox/flox^ (Stat3^fl/fl^) mice were infected with Aspergillus fumigatus in an aerosol chamber, and the weight, activity, appearance, and respiratory rate of the mice were monitored daily for 21 days to evaluate their survival. *Aspergillus* infection was confirmed by lung fungal culture counts, histology, and a galactomannan test. Cytokines were measured at 3 days postinfection in bronchoalveolar lavage (BAL) fluid and serum. Immunosuppressed CD4*^Stat3^*^−/−^ mice began succumbing to infection by day 4, and by day 7, only 30% of mice survived. Immunosuppressed Stat3^fl/fl^ mice started to succumb to the disease on day 5, and 40% of mice remained by day 7. The nonimmunosuppressed control Stat3^fl/fl^ and CD4*^Stat3^*^−/−^ mice maintained their weight over the study period, without any evidence of infection by A. fumigatus by histology. In the BAL fluid, tumor necrosis factor alpha (TNF-α), interleukin-6 (IL-6), interferon gamma (IFN-γ), IL-17A, and IL-22 levels were elevated in Stat3^fl/fl^ immunosuppressed mice compared to immunosuppressed CD4*^Stat3^*^−/−^ mice at 3 days postinfection. STAT3 in CD4^+^ T cells modulates the production of cytokines in the IL-17 pathway in immunosuppressed mice. However, it has no meaningful effect on the clearance of *Aspergillus* or the concomitant increase in susceptibility to *Aspergillus* infection.

## Abstract (precis)

We aimed to determine whether T cell-specific STAT3 deletion influences the immune response to *Aspergillus* in the immunosuppressed context in CD4*^Stat3^*^−/−^ mice. Immunosuppressed and nonimmunosuppressed CD4*^Stat3^*^−/−^ mice and littermate Stat3^flox/flox^ (Stat3^fl/fl^) mice were infected with Aspergillus fumigatus in an aerosol chamber, and the weight, activity, appearance, and respiratory rate of the mice were monitored daily for 21 days to evaluate their survival. *Aspergillus* infection was confirmed by lung fungal culture counts, histology, and a galactomannan test.

## INTRODUCTION

Invasive aspergillosis (IA) is a common opportunistic fungal infection caused by *Aspergillus* species in immunocompromised patients. An effective immune response against *Aspergillus* depends on the coordination of innate and adaptive immunity (1). Alveolar macrophages and neutrophils recognize conidia and hyphae via pathogen recognition receptors (PPRs) such as Dectin-1, Toll-like receptor 2 (TLR2), and TLR4 and secrete cytokines to inform the adaptive immune response (2). Following fungal recognition, *Aspergillus* antigens are processed by dendritic cells and presented to naive CD4^+^ T cells to induce their differentiation into T helper 1 (Th1) or Th17 cells; both subsets promote fungal clearance (3).

During *Aspergillus* infection, the initial innate immune response is mediated by pathogen recognition receptors such as Dectin-1 and TLRs, which recognize *Aspergillus* conidia, initiating the activation of Th1 and Th17 cells (4, 5). Adaptive immunity develops when dendritic cells present fungal peptides to *Aspergillus*-specific CD4^+^ naive T cells and induce their differentiation into Th1 and Th17 cells. The transcription factor signal transducer and activator of transcription 3 (STAT3) is activated by interleukin-6 (IL-6) and plays an essential role in Th17 differentiation by activating retinoic acid receptor (RAR)-related orphan receptor gamma (RORγ), a nuclear receptor selectively expressed in Th17 cells, to induce the expression of IL-17 (6). Following their differentiation, Th17 cells can recruit and activate neutrophils at the site of infection, induce proinflammatory cytokines and chemokines, as well as secrete IL-22, which initiates the production of antimicrobial peptides (4, 5, 7, 8).

While Th17 cells are crucial in the host response to *Aspergillus* infection, the role of STAT3 in IA is unclear. In peripheral blood from IA patients with leukemia, we previously reported the decreased phosphorylation of STAT3 in circulating CD4^+^ T cells in response to IL-6 (9). *In vitro*, IL-6 does not increase IL-17 production in peripheral blood mononuclear cell culture supernatants from patients with IA. Still, it increases IL-17 production in supernatants from controls, suggesting that dysregulation of the IL-6/STAT3 axis disrupts Th17 cell differentiation and IL-17 production in IA patients (9).

In humans, a loss-of-function mutation in STAT3 has been identified as the molecular basis for autosomal dominant hyper-immunoglobulin E (IgE) syndrome (HIES), a primary immunodeficiency characterized by elevated serum IgE, rashes, and recurrent bacterial infections of the skin and lung (10). HIES patients are particularly susceptible to late-onset invasive fungal diseases like aspergillosis, especially when there is previous lung damage (11). According to a French national survey of 60 patients, 22% of HIES patients developed aspergillosis (12). Recently, Danion et al. showed that patients with STAT3 deficiency, particularly those with aspergillosis, produce lower concentrations of the adaptive cytokines interferon gamma (IFN-γ), IL-17, and IL-22 (13). STAT3-deficient patients also had lower levels of IFN-γ- and IL-17-producing CD4^+^ T cells. However, they observed no defects in the innate immune response in STAT3-deficient patients (13).

Although the role of STAT3 deficiency has been explored in HIES, its role in the setting of immunosuppression has not been well documented. All individuals with chemotherapy during leukemia develop prolonged neutropenic fever, and their duration of neutropenia ranges from 21 to 28 days. Yet only 8 to 24% of leukemia patients with neutropenic fever develop IA in the absence of mold-directed antifungal prophylaxis (14, 15). We hypothesized that constitutive STAT3 deficiency may increase the susceptibility to the development of IA in immunosuppressed patients with neutropenia, resulting in higher mortality rates. We chose to investigate this in a murine model of IA. We sought to determine whether the loss of STAT3 promotes the development of IA in immunosuppressed mice. Constitutive STAT3 deletion causes embryonic lethality. Due to the role that STAT3 plays in orchestrating CD4^+^ T cell differentiation to Th17 cells, we generated mice that had STAT3 deleted in CD4^+^ T cells only.

## RESULTS

**FIG 1:**
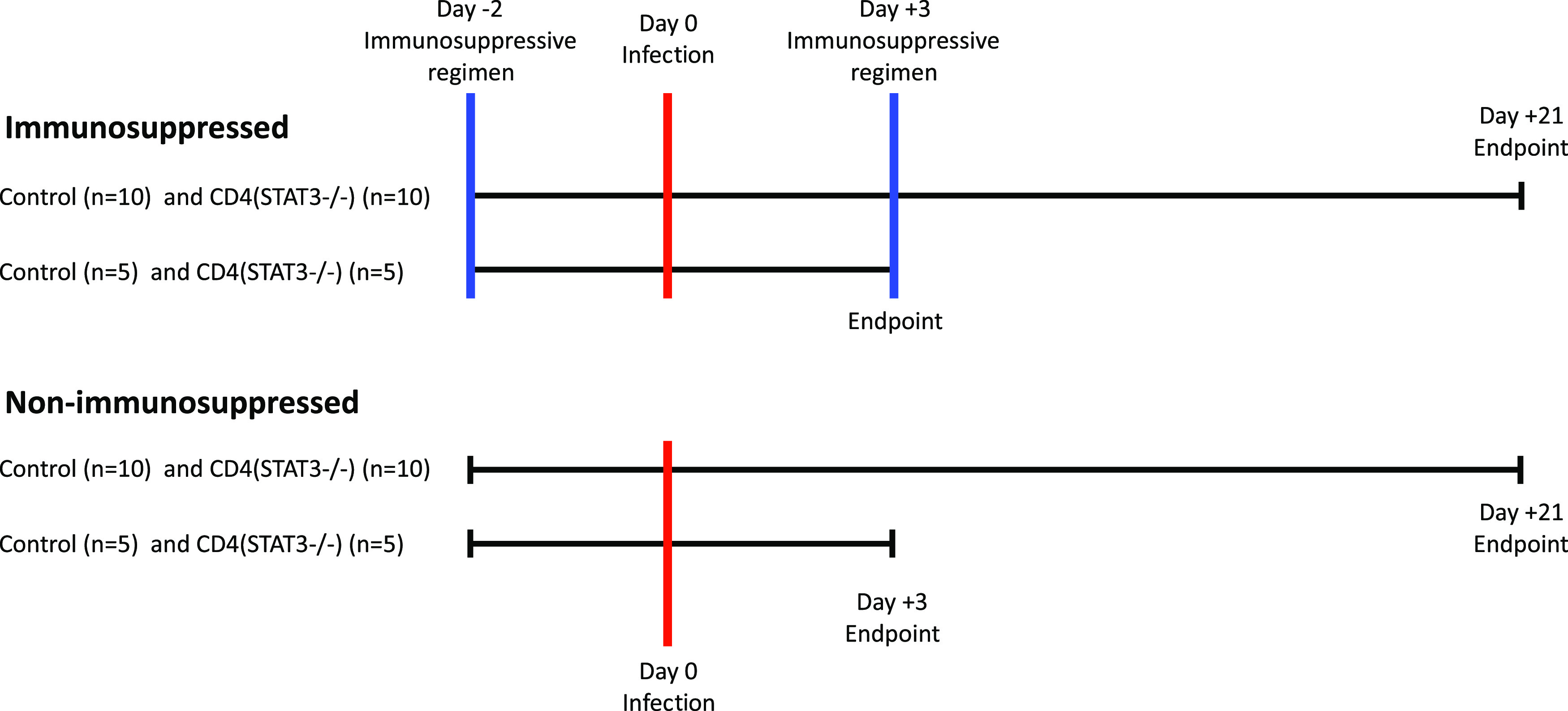
Schematic representation of the experimental model. Eight- to ten-week-old control mice (*n* = 30) and CD4*^Stat3^*^−/−^ mice (*n* = 30) were assigned to the immunosuppressed or nonimmunosuppressed group. The mice in the immunosuppressed group received an immunosuppressive regimen (cortisone acetate [250 mg/kg] and cyclophosphamide [250 mg/kg]) on day −2 and day +3. Mice were infected with A. fumigatus in an inhalation chamber. A subset of mice (*n* = 5/group) was sacrificed on day 3 postinfection. The remaining mice (*n* = 10/group) were monitored and sacrificed when moribund or at day 21 postinfection.

Prolonged neutropenia and corticosteroid use remain some of the most critical risk factors for IA. In the present study, we immunosuppressed mice by administrating cortisone acetate and cyclophosphamide to induce neutropenia. To mimic IA, we infected mice with Aspergillus fumigatus in an inhalation chamber. A schematic representation of the experimental model is depicted in Fig. 1. Complete blood counts (CBCs) confirmed that mice were neutropenic at the time of infection and remained neutropenic up to 1 week after infection (see Fig. S1 in the supplemental material).

**FIG 2:**
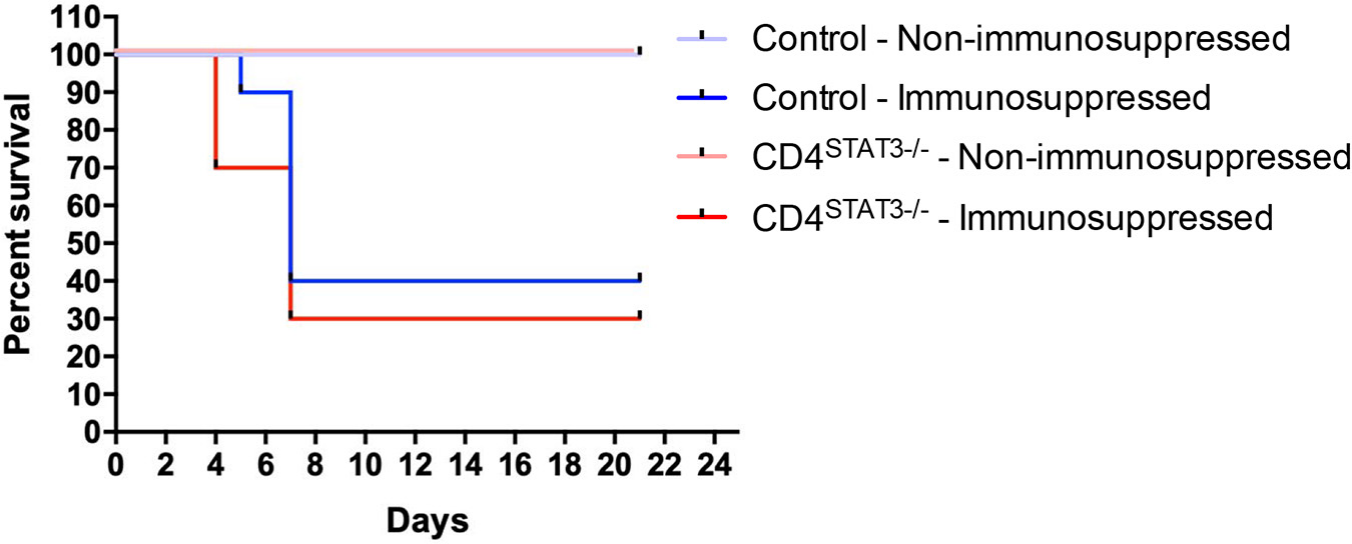
Survival of control and CD4*^Stat3^*^−/−^ nonimmunosuppressed and immunosuppressed mice after *Aspergillus* infection (*n* = 10/group).

Early in infection, the survival rate was lower for immunosuppressed CD4*^Stat3^*^−/−^ mice than for control mice (Stat3^fl/fl^) (Fig. 2); the difference was not statistically significant. At 4 days postinfection (dpi), the survival rate of immunosuppressed CD4*^Stat3^*^−/−^ mice was 70%, whereas it was 100% for immunosuppressed control mice. Immunosuppressed control mice began succumbing to infection at 5 dpi, and by 7 dpi, 60% of mice had died. In immunosuppressed CD4*^Stat3^*^−/−^ mice, 70% of mice had been killed at 7 dpi. All nonimmunosuppressed mice survived for the duration of the study.

**FIG 3:**
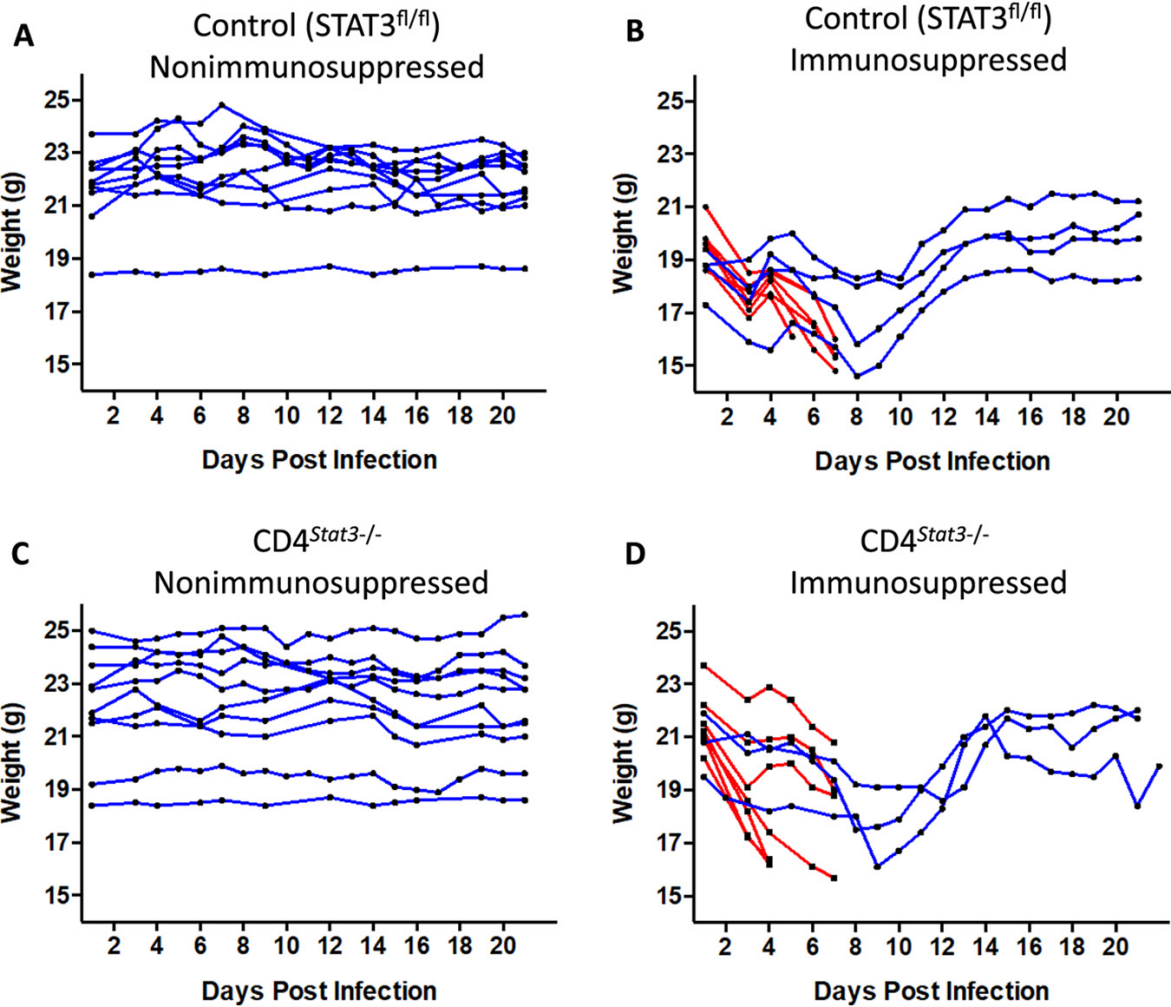
Total body weights of each group of mice over the course of 21 days. (A) Control nonimmunosuppressed (*n* = 10); (B) control immunosuppressed (*n* = 10); (C) CD4*^Stat3^*^−/−^ nonimmunosuppressed (*n* = 10); (D) CD4*^Stat3^*^−/−^ immunosuppressed (*n* = 10). Mice that succumbed to infection and were euthanized before the end of the study are highlighted in red.

Nonimmunosuppressed mice in both groups maintained their weight throughout the study (Fig. 3A and A). All immunosuppressed mice lost weight after infection (Fig. 3B and D). Control immunosuppressed mice that succumbed to disease lost 16 to 22% of their body weight, and immunosuppressed CD4*^Stat3^*^−/−^ mice lost 16 to 25% of their body weight. The mice that did not succumb to infection were able to regain their weight by 8 dpi.

**FIG 4:**
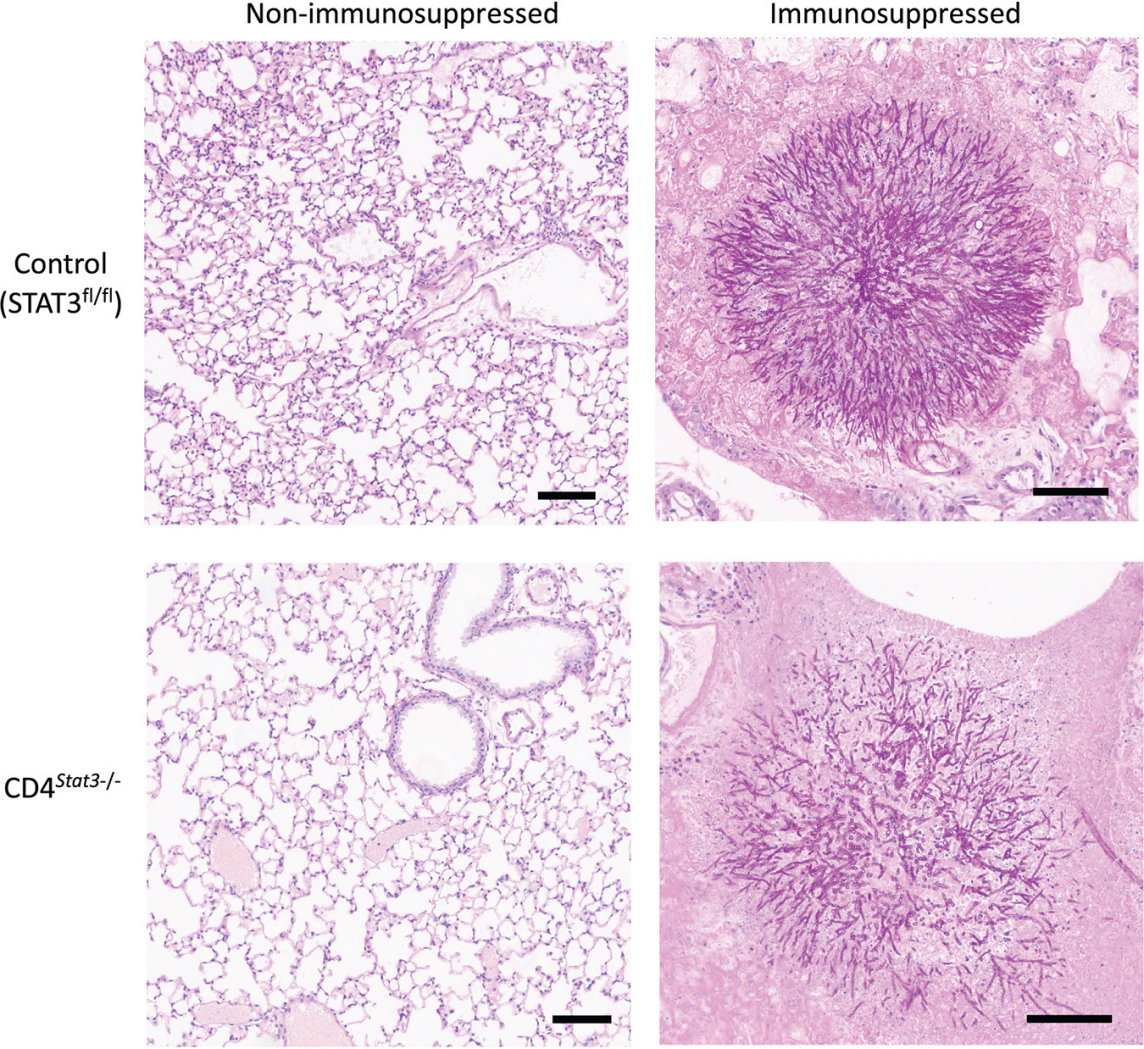
PAS staining of lungs from nonimmunosuppressed mice that survived the 3-week study and immunosuppressed mice that succumbed to infection. Bars, 100 μm.

The lungs from mice that did not survive had multiple large nodules of germinated conidia and hyphae consistent with A. fumigatus when stained with periodic acid-Schiff (PAS) stain, confirming the presence of the fungi in the lungs (Fig. 4). No fungi were present in the lungs from nonimmunosuppressed mice that survived for the duration of the study. Bronchoalveolar lavage (BAL) fluid from mice that succumbed to infection all had a galactomannan (GM) index of >1.

**FIG 5:**
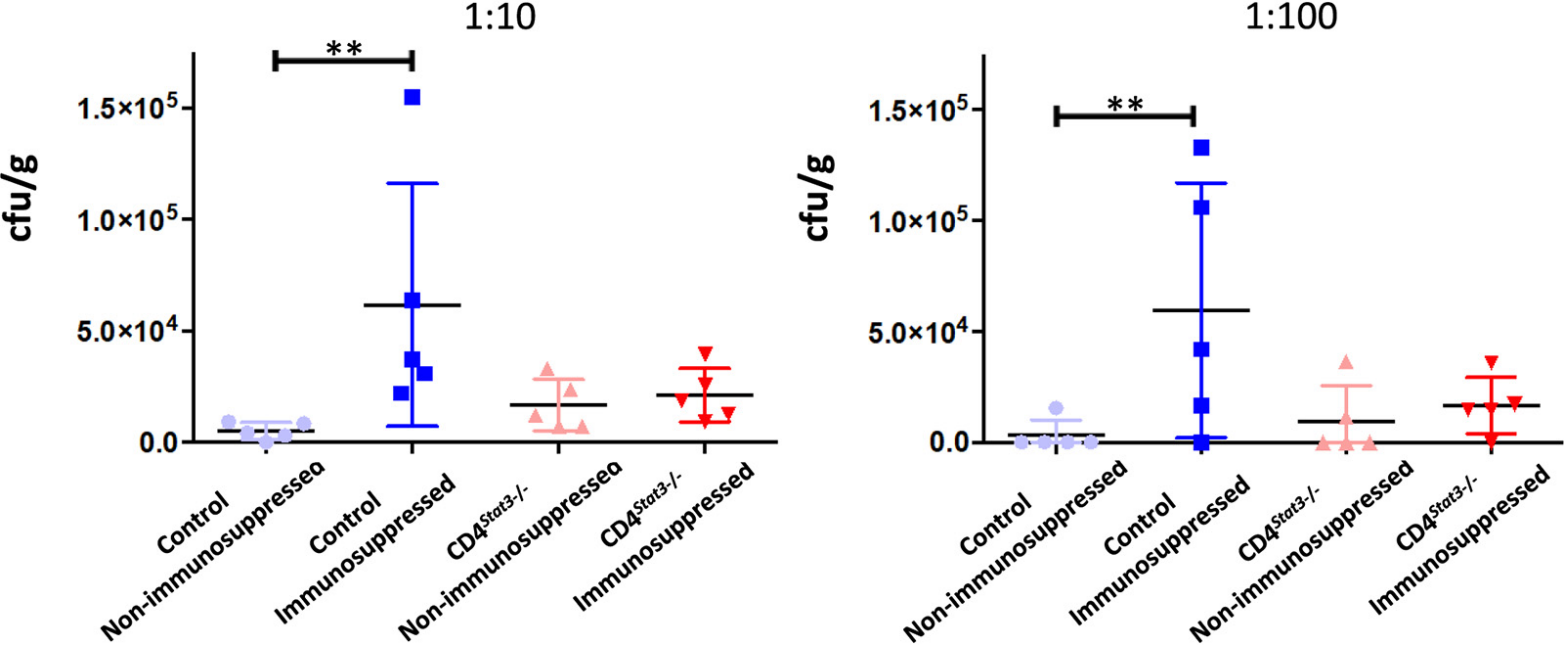
Homogenized lung culture counts at 3 days postinfection (*n* = 5/group). **, *P* < 0.01.

The lung fungal burden at 3 dpi was significantly higher in immunosuppressed control mice than in nonimmunosuppressed controls (*P* = 0.0079) (Fig. 5). However, there was no difference in the fungal burdens between CD4*^Stat3^*^−/−^ nonimmunosuppressed and immunosuppressed mice. In all groups, brain fungal burden, quantified by plating homogenized brain on potato dextrose agar (PDA) plates, was not observed (data not shown).

**FIG 6:**
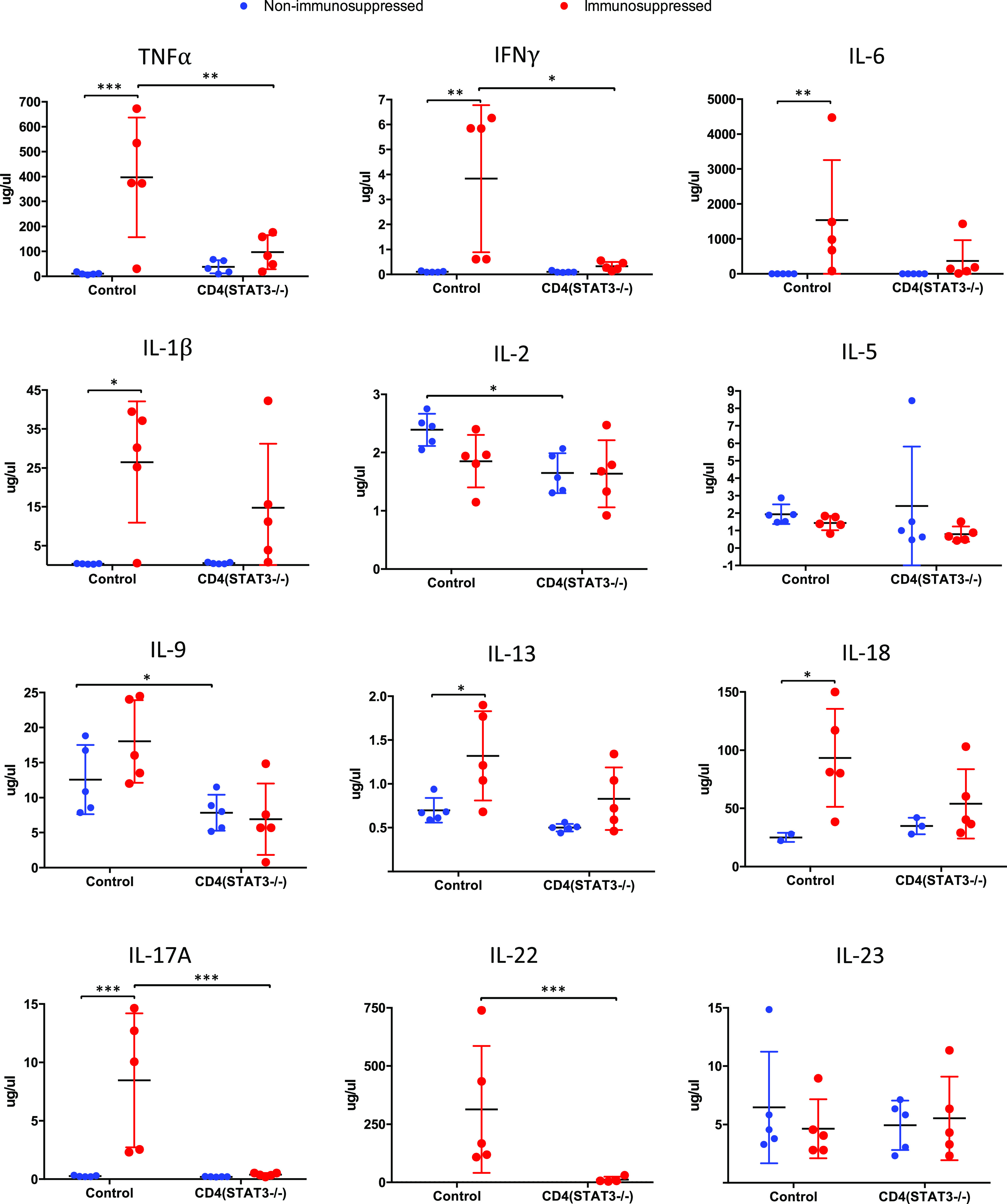
Cytokine levels in BAL fluid of control and CD4*^Stat3^*^−/−^ mice at 3 days postinfection (*n* = 5/group). *, *P* < 0.05; **, *P* < 0.01; ***, *P* < 0.001.

CD4^+^ T cells play a critical role in mounting an immune response to A. fumigatus. The differentiation of CD4^+^ T cells depends on the cytokine milieu and specific transcription factors such as STAT3. We measured cytokines in BAL fluid and plasma of mice at 3 dpi to define the impact of STAT3 deletion in CD4^+^ T cells on cytokine expression (Fig. 6). Tumor necrosis factor alpha (TNF-α), IFN-γ, IL-6, IL-1β, IL-13, IL-18, and IL-17 levels were significantly higher in BAL fluid of immunosuppressed control mice than in nonimmunosuppressed controls. While TNF-α, IFN-γ, IL-2, IL-17, and IL-22 levels were significantly higher in immunosuppressed control mice than in immunosuppressed CD4*^Stat3^*^−/−^ mice (*P* < 0.05), IL-2 and IL-9 levels were higher in nonimmunosuppressed control mice than in nonimmunosuppressed CD4*^Stat3^*^−/−^ mice. IL-10 and IL-27 expression levels were below the detectable range.

**FIG 7:**
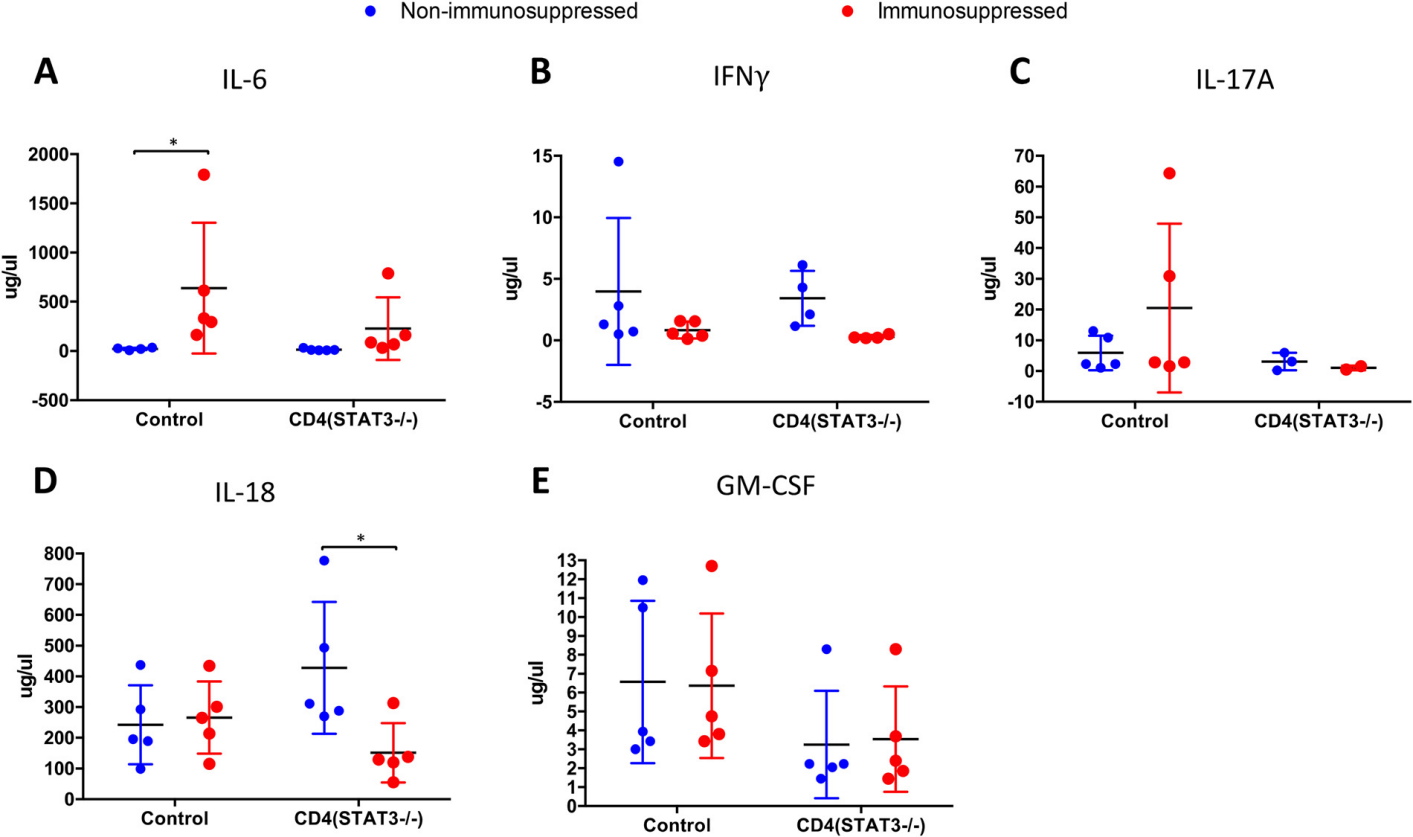
Cytokine levels in plasma of control and CD4*^Stat3^*^−/−^ mice at 3 days postinfection (*n* = 5/group). *, *P* < 0.05.

At 3 dpi, most cytokines were not detected in the plasma (Fig. 7). IL-6 was elevated in the plasma of immunosuppressed control mice compared to nonimmunosuppressed controls. No significant differences in the levels of IFN-γ, IL-17A, and granulocyte-macrophage colony-stimulating factor (GM-CSF) were observed. IL-18 levels were higher in plasma from nonimmunosuppressed CD4*^Stat3^*^−/−^ mice than in plasma from immunosuppressed CD4*^Stat3^*^−/−^ mice (*P* = 0.0359).

## DISCUSSION

While numerous risk factors for IA have been identified, IA develops in only a minority of patients, thereby suggesting the existence of as-yet-unidentified host risk factors that influence susceptibility to *Aspergillus*. Th17 cells are crucial during A. fumigatus infection in inducing an inflammatory response, and their differentiation depends on IL-6 signaling via the transcription factor STAT3, which activates the expression of cytokines such as IL-17 and IFN-γ (16). Whether STAT3 is essential for the host response to A. fumigatus is unknown. In this study, we used a mouse model of IA to examine the susceptibility of CD4*^Stat3^*^−/−^ mice to IA and measured cytokine expression to evaluate whether the cytokine milieu in the BAL fluid and plasma is impacted by the loss of STAT3 in CD4^+^ T cells.

The deletion of STAT3 in CD4^+^ T cells did not affect the survival rate of mice with IPA in the long term. Early in the infection, 30% of CD4*^Stat3^*^−/−^ mice had succumbed to infection; however, over the course of 3 weeks, the overall survival rate compared to control mice was not significantly different. The differences were small, which would suggest that a large number of additional animals would be needed to demonstrate an effect. Our data suggest that STAT3 in CD4^+^ T cells is not essential for the host immune response against A. fumigatus, and STAT3 deletion in CD4^+^ T cells does not lead to a significantly greater reduction in survival. This may suggest that the degree of immunosuppression was too high, masking the role of STAT3 in CD4-expressing cells. This could be evaluated in follow-up work by reducing the dose of cyclophosphamide. Additionally, follow-up studies using lower concentrations of *Aspergillus* conidia to infect mice may help delineate differences between immunosuppressed control and CD4*^Stat3^*^−/−^ mice.

STAT3 signaling in T cells is a mandatory requirement for Th17 cell differentiation in autoimmune models as well as for the maintenance of endogenous Th17 cells in CD4*^Stat3^*^−/−^ mice (17, 18). During autoimmune pneumonitis, CD4*^Stat3^*^−/−^ mice have a complete loss of RORγ, which is a crucial characteristic of Th17 cells (17). Due to these findings, along with the results showing that Dectin-1 signaling enables Th17 differentiation during A. fumigatus infection and higher susceptibility to IA in humans with STAT3 mutations, we anticipated that CD4*^Stat3^*^−/−^ mice would be more susceptible to IA. However, the role of Th17 cells and STAT3 during *Aspergillus* infection appears to be more complicated. Inflammation, although necessary to initiate the host immune response against *Aspergillus*, can also impair fungal clearance when not resolved. Prolonged inflammation is causally linked to chronic and autoimmune diseases and has been shown to result in defective fungal clearance (18).

Contrary to the proposed protective role of Th17 cells during aspergillosis, Zelante et al. have shown that the Th17 pathway can cause prolonged inflammation that exacerbates A. fumigatus and Candida albicans infections (20). IL-23, which synergizes with IL-6 to activate STAT3 and promote Th17 differentiation, acts as a negative regulator for Th1-meditated immune resistance to A. fumigatus. Those researchers found that IL-23-deficient mice had lower fungal burdens; blockade of IL-23 in control mice also lowered the fungal burden, increased IFN-γ-positive (IFN-γ^+^) Th1 cells, and decreased Th17 cell populations. Together, their findings suggest that the IL-23/Th17/IL-17 pathway promotes exacerbated inflammation and increased susceptibility to fungal infection by inhibiting Th1 immunity (20).

In the present study, infected CD4*^Stat3^*^−/−^ mice had a lower fungal burden in the lungs than that reported in a previous study (19). We observed significantly decreased levels of IL-6 and IL-17A in CD4*^Stat3^*^−/−^ mice. Taken in the context of Th17 cells contributing to exacerbated inflammation that allows uncontrollable fungal growth, CD4^+^ T cell-specific deletion of STAT3 appears to impair Th17 cell differentiation, as seen by the reduction of Th17-secreted cytokines in the BAL fluid, such as IL-17, TNF-α, IL-1β, granulocyte colony-stimulating factor (G-CSF), and IL-22. The role of Th17 cells during *Aspergillus* infection remains contentious. Several studies have shown that Th17 cells are essential for triggering neutrophil recruitment to the site of infection. Neutrophils can enhance the production of radical oxygen species (ROS), proteolytic enzymes, and antimicrobial peptides, which all aid in eliminating fungi. Despite the critical roles that Th17 cells play, prolonged Th17 activation appears to be detrimental for the host in many immune-mediated diseases such as psoriasis, rheumatoid arthritis, and multiple sclerosis (21). How Th17 cell activation is balanced during *Aspergillus* infection remains unclear.

High levels of IL-23 are secreted by dendritic cells for the induction of Th17 cells (5). In our study, IL-23 levels do not appear to be affected by STAT3 deletion, suggesting normal dendritic cell function in CD4*^Stat3^*^−/−^ mice. In control (Stat3^fl/fl^) immunosuppressed mice, we observed elevated levels of TNF-α, IFN-γ, IL-6, and IL-17A in the BAL fluid. This is consistent with what others have reported in immunosuppressed mice during IA (22). The mortality rate of 60% in control immunosuppressed mice is also compatible with what has been previously reported in the literature (23).

Due to STAT3’s essential role during early embryonic development, the constitutive deletion of STAT3 is embryonic lethal (24). We chose to investigate STAT3 in T cells; however, the STAT3 mutations observed in HIES patients, who are highly susceptible to IA, are not limited to a specific cell type. STAT3 variation affects multiple cell lineages in HIES patients, such as B cells, macrophages, and dendritic cells, along with T cells (25). In B cells, STAT3 deficiency impairs the T cell-dependent IgG response. In macrophages, the lack of STAT3 affects IL-10 production, which plays a crucial role in attenuating proinflammatory cytokines during infections (26, 27). This could indicate that increased susceptibility to A. fumigatus could be augmented by STAT3 deficiency in multiple types of immune cells. Thus, CD4^+^ T cell deficiency is not enough to show increased susceptibility.

We chose to infect mice using an inhalation chamber to mimic real-life pulmonary infection with A. fumigatus. This is a potential limitation as it results in heterogeneity in the numbers of conidia that are initially delivered to each mouse. Additionally, our sample size calculation implied that 90% of CD4*^Stat3^*^−/−^ mice will get the disease, compared to 15% of CD4*^Stat3^*^+/+^ mice. This gave the study 80% power to detect a difference. It may be possible that our data assumption may not be accurate and may be underpowered. However, in the absence of existing data in immunocompromised hosts, this appears to be a reasonable estimate.

In summary, our data highlight the critical role of STAT3 in the stimulation of the Th17 pathway. However, STAT3 deficiency in CD4^+^ T cells was not associated with increased mortality or a higher burden of disease in mice. Future studies with more significant numbers of additional animals and various degrees of immunosuppression may be required to decipher the exact role of STAT3 deficiency in CD4^+^ T cells in susceptibility to aspergillosis in immunosuppressed mice.

## MATERIALS AND METHODS

### Ethical approval.

All animal protocols for this study were approved by the University Health Network (UHN) Animal Care Committee (animal utilization protocol 5634) and were performed according to the Animals for Research Act, the guidelines and policies of the Canadian Council on Animal Care (https://www.ccac.ca/en/standards/guidelines/), and institutional policies.

### *Aspergillus* culture conditions.

Aspergillus fumigatus strain AF 293 (ATCC MYA-4609) was used to infect mice. Ten days before the experiment, potato dextrose agar (PDA) plates were inoculated with A. fumigatus and incubated at 37°C. On the day of the experiment, conidia were harvested by washing plates with sterile phosphate-buffered saline (PBS) with 0.1% (vol/vol) Tween 80 (PBST) and concentrating conidia by centrifugation. The conidial suspension concentration was determined by counting 1:1,000 and 1:10,000 dilutions in PBST using a hemacytometer, and the inoculum was diluted to a target concentration of 1 × 10^9^ conidia/ml.

### Mouse breeding.

Mice with a deletion of *Stat3* in CD4 T cells (CD4*^Stat3^*^−/−^ mice) were generated by breeding CD4 Cre mice [stock Tg(Cd4-Cre)1Cwi/BfluJ, stock no. 017336] with Stat3^fl/fl^ mice (B6.129S1-Stat3tm1Xyfu/J, stock no. 016923), obtained from Jackson Laboratories. Littermate Stat3^fl/fl^ mice were used as controls in the experiments. Each mouse was genotyped to ensure that Stat3^fl/fl^ and CD4*^Stat3^*^−/−^ were used for the experiment.

### Animal model for invasive aspergillosis.

Eight- to ten-week-old Stat3^fl/fl^ mice and CD4*^Stat3^*^−/−^ mice were infected with A. fumigatus in an acrylic inhalation chamber. Before the infection, mice were randomly assigned to the immunosuppressed or nonimmunosuppressed group (*n* = 12/group). Mice were immunosuppressed 2 days before infection and at 3 days postinfection (dpi) with cortisone acetate (250 mg/kg of body weight) and cyclophosphamide (250 mg/kg) to induce neutropenia. Immunosuppressed mice were also administered ceftazidime (50 mg/kg) subcutaneously daily to prevent bacterial infections. Immunosuppression was confirmed by performing complete blood counts (CBCs) before immunosuppression, on the day of infection, and at 7 dpi. Mice were then infected with 12 ml of 1 × 10^9^ conidia/ml using a nebulizer for 1 h. Two mice from each group were euthanized within 1 h after infection; their lungs were homogenized, serially diluted, and plated; and CFU were counted 24 h later to ensure infectivity. Mice were weighed daily and monitored for activity, appearance, posture, respiratory rate, and hydration. Five mice in each group were sacrificed at 3 dpi to quantify fungal burdens in their lungs and brains and to measure plasma cytokines during early infection. The remaining mice were monitored daily for 21 days and sacrificed when moribund. At the endpoint, the lungs, brain, BAL fluid, and plasma of the mice were collected for future analysis. A midline incision was made through the skin and abdomen, the diaphragm and ribcage were cut, and cardiac puncture was performed to collect blood. To perform the cardiac puncture, a 25-gauge needle was used with a 1-ml syringe. Next, lungs were harvested by clamping the trachea and removing both the left and right lobes. Mouse brains were harvested by cutting upward from the brain stem along the sagittal suture. The two halves of the skull were removed, and the brain was carefully harvested.

### Fungal burden.

Tissues were weighed and homogenized in 5 ml of sterile PBS with Tween 20 using a gentleMACS dissociator to determine the fungal burdens in lungs and brains. Homogenized samples were diluted 1:10 and 1:100, and 10 μl of the dilution was plated onto PDA plates. The plates were incubated at 37°C, and CFU were counted 24 h after incubation.

### Histology.

Lungs were inflated with 1 ml of formalin using a syringe inserted into the trachea. After inflation, the trachea was tied to prevent leakage of formalin. The lungs were submerged in formalin for 24 h and then transferred to 70% ethanol before embedding in paraffin. Once embedded in paraffin, lungs were sectioned at 8 μm and stained with periodic acid-Schiff (PAS) to visualize fungi.

### GM Platelia *Aspergillus* assay.

Galactomannan in mouse BAL fluid was detected using a Platelia *Aspergillus* enzyme immunoassay (Bio-Rad Laboratories, Redmond, WA). Each sample was tested in duplicate, alongside a negative-control, a positive-control, and a cutoff control sample provided by the manufacturer. Positive BAL fluid was defined as a sample with an optical density (OD) index of >1. The index of each sample was calculated to determine the presence or absence of GM. The index of a sample was the OD value of the sample divided by the mean OD of cutoff control samples.

### Cytokine Bioplex.

Cytokine levels were measured in mouse BAL fluid and serum using the Invitrogen Th1/Th2/Th9/Th17/Th22/regulatory T cell (Treg) cytokine 17-plex mouse ProcartaPlex panel (Thermo Fisher Scientific). The following cytokines were measured: IFN-γ, IL-12p70, IL-13, IL-1β, IL-2, IL-4, IL-5, IL-6, TNF-α, GM-CSF, IL-18, IL-10, IL-17A, IL-22, IL-23, IL-27, and IL-9. Each sample was tested in duplicate.

### Statistical analysis.

Survival curves were analyzed using the log rank (Mantel-Cox) test. BAL fluid and plasmatic cytokine data were analyzed using two-way analysis of variance (ANOVA) and Sidak’s multiple-comparison test. All data analysis was performed with GraphPad Prism 6.

## Supplementary Material

Supplemental file 1
