# Supplementary material for: Evaluating the Role of STAT3 in CD4+ T Cells in Susceptibility to Invasive Aspergillosis
Source: Infect Immun. 2021 Apr 16;89(5):e00035-21. doi: 10.1128/IAI.00035-21 (PMC8091102; doi:10.1128/IAI.00035-21)

Supplementary Figure 1: White blood cells (WBC) and neutrophils counts were performed 7 days before infection, on day of infection and 7 days post infection. A) White blood cell counts represented in cells/L for control and CD4$^{Stat3-/-}$ mice. B) Neutrophil counts represented cells/L for control and CD4$^{Stat3-/-}$ mice.

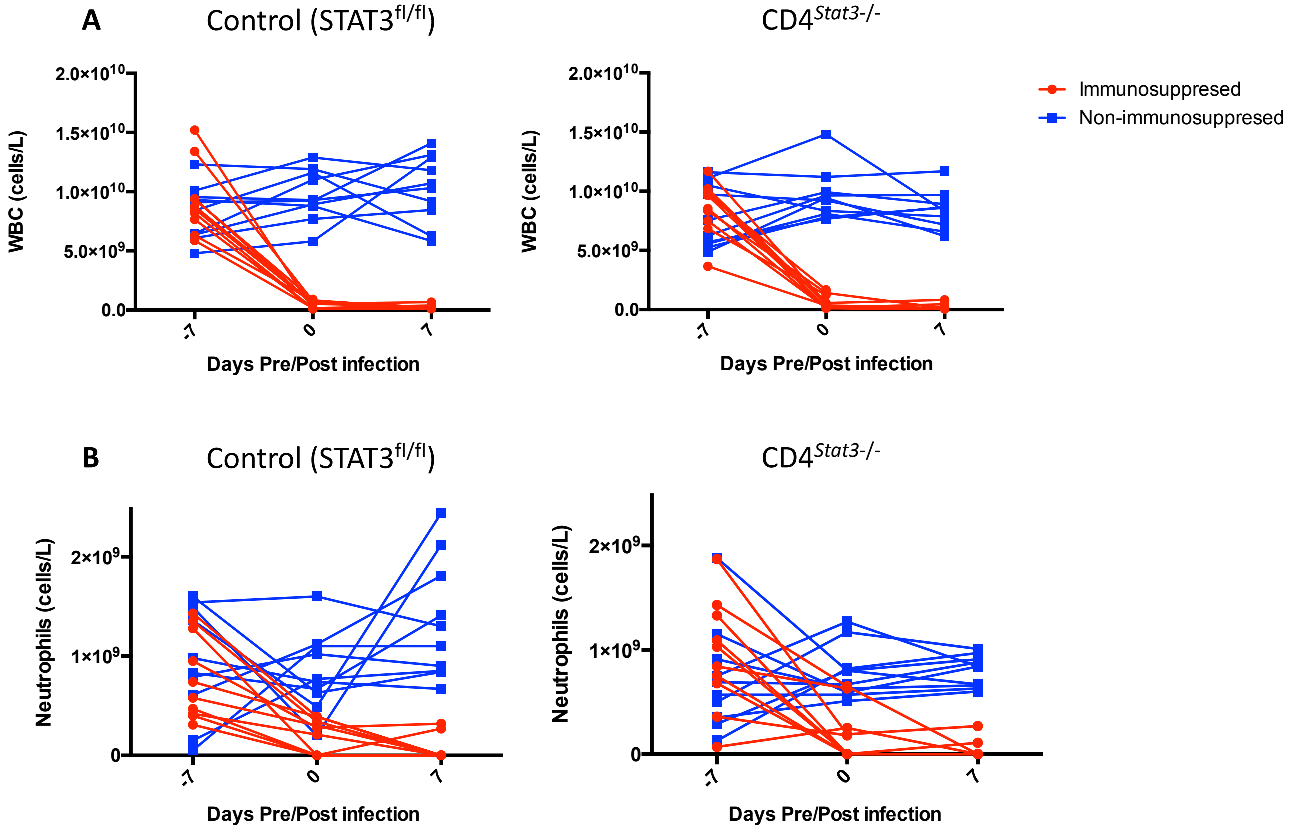

Supplement: Supplemental file 1 [file IAI.00035-21-s0001.pdf]
